# Crystal structure of *N*′-di­phenyl­methyl­idene-5-methyl-1*H*-pyrazole-3-carbo­hydrazide

**DOI:** 10.1107/S2056989015020071

**Published:** 2015-10-28

**Authors:** Khalid Karrouchi, M’hammed Ansar, Smaail Radi, Mohamed Saadi, Lahcen El Ammari

**Affiliations:** a Laboratory of Medicinal Chemistry, Faculty of Medicine and Pharmacy, University Mohammed V, Rabat, Morocco; b LCAE, Department of Chemistry, Faculty of Sciences, University Mohamed I, Oujda, Morocco; c Laboratoire de Chimie du Solide Appliquée, Faculté des Sciences, Université Mohammed V, Avenue Ibn Battouta, BP 1014, Rabat, Morocco

**Keywords:** crystal structure, pyrazole derivatives, biological activity, agrochemical applications, pharmaceutical applications

## Abstract

In the title compound, C_18_H_16_N_4_O, the planes of the phenyl rings are approximately perpendicular to each other [dihedral angle = 78.07 (8)°] and form dihedral angles of 56.43 (8) and 24.59 (8)° with the pyrazole ring. In the crystal, mol­ecules are linked by N—H⋯O hydrogen bonds to form one-dimensional chains parallel to the [010] direction.

## Related literature   

For the biological activities of pyrazole derivatives, see: Zhang *et al.* (2015 ▸); Özdemir *et al.* (2015 ▸); El-Sabbagh *et al.* (2009 ▸); Farag *et al.* (2010 ▸); Karrouchi *et al.* (2014 ▸); Mert *et al.* (2014 ▸); Alegaon *et al.* (2014 ▸). For the applications in agrochemical and pharmaceutical industries of pyrazole derivatives, see: Patel *et al.* (2004 ▸). For the structure of a related compound, see: Karrouchi *et al.* (2013 ▸).
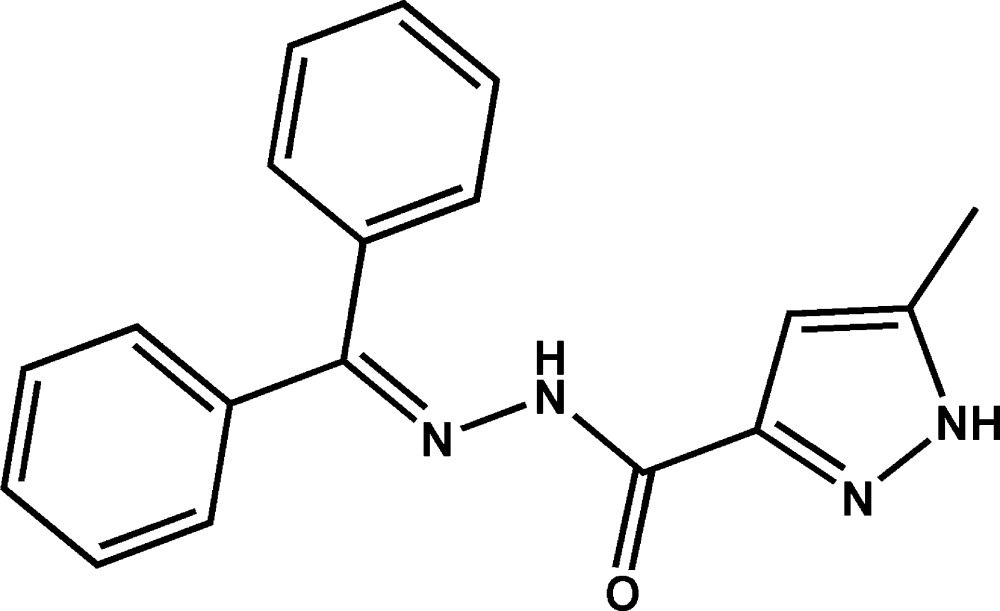


## Experimental   

### Crystal data  

C_18_H_16_N_4_O
*M* *_r_* = 304.35
Orthorhombic 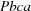

*a* = 11.0299 (2) Å
*b* = 14.1131 (2) Å
*c* = 20.2211 (3) Å
*V* = 3147.74 (9) Å^3^
*Z* = 8
Mo *K*α radiation
μ = 0.08 mm^−1^
*T* = 296 K
0.40 × 0.32 × 0.25 mm

### Data collection  

Bruker X8 APEX diffractometer
31259 measured reflections
3766 independent reflections
3117 reflections with *I* > 2σ(*I*)
*R*_int_ = 0.029

### Refinement  

*R*[*F* ^2^ > 2σ(*F* ^2^)] = 0.047
*wR*(*F* ^2^) = 0.136
*S* = 1.04
3766 reflections
208 parameters
H-atom parameters constrained
Δρ_max_ = 0.33 e Å^−3^
Δρ_min_ = −0.26 e Å^−3^

### 

Data collection: *APEX2* (Bruker, 2009 ▸); cell refinement: *SAINT-Plus* (Bruker, 2009 ▸); data reduction: *SAINT-Plus*; program(s) used to solve structure: *SHELXS97* (Sheldrick, 2008 ▸); program(s) used to refine structure: *SHELXL2014* (Sheldrick, 2015 ▸); molecular graphics: *ORTEP-3 for Windows* (Farrugia, 2012 ▸); software used to prepare material for publication: *PLATON* (Spek, 2009 ▸) and *publCIF* (Westrip, 2010 ▸).

## Supplementary Material

Crystal structure: contains datablock(s) I. DOI: 10.1107/S2056989015020071/rz5174sup1.cif

Structure factors: contains datablock(s) I. DOI: 10.1107/S2056989015020071/rz5174Isup2.hkl

Supporting information file. DOI: 10.1107/S2056989015020071/rz5174Isup3.cml

**Fig. 1.**
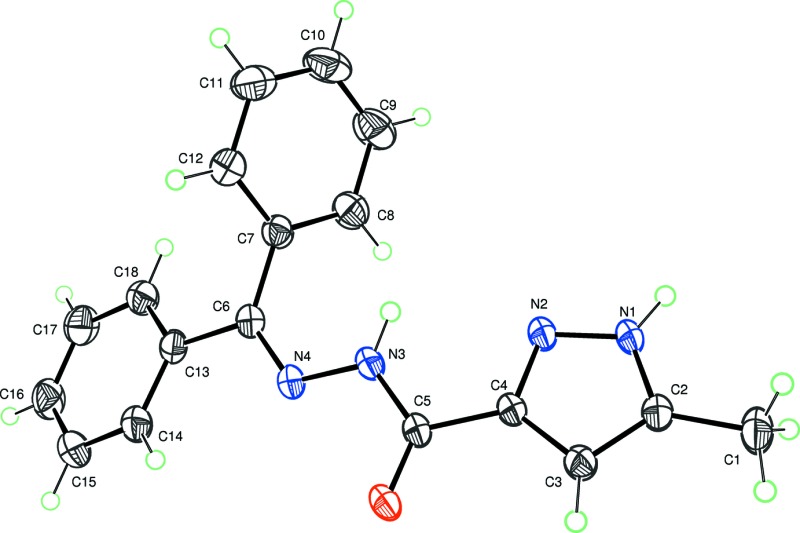
The molecular structure of the title compound with displacement ellipsoids drawn at the 50% probability level. H atoms are represented as small circles.

**Fig. 2.**
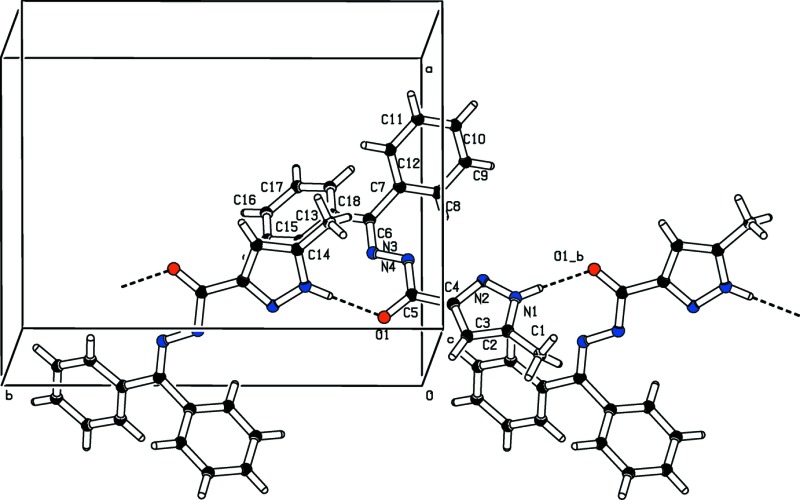
Partial crystal packing of the title compound, showing molecules linked by N–H···O hydrogen bonds (dashed lines) into a chain parallel to the b axis.

. DOI: 10.1107/S2056989015020071/rz5174fig1.tif
The mol­ecular structure of the title compound with displacement ellipsoids drawn at the 50% probability level. H atoms are represented as small circles.

b . DOI: 10.1107/S2056989015020071/rz5174fig2.tif
Partial crystal packing of the title compound, showing mol­ecules linked by N–H⋯O hydrogen bonds (dashed lines) into a chain parallel to the *b* axis.

CCDC reference: 1432912

Additional supporting information:  crystallographic information; 3D view; checkCIF report

## Figures and Tables

**Table 1:** Hydrogen-bond geometry (, )

*D*H*A*	*D*H	H*A*	*D* *A*	*D*H*A*
N1H1*N*O1^i^	0.86	2.02	2.8740(15)	172

Symmetry code: (i) 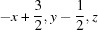.

## supplementary crystallographic information

### S1. Comment

Compounds containing the pyrazole moiety are known to exhibit a wide range of biological properties such as anticancer (Zhang *et al.*, 2015), anticonvulsant (Özdemir *et al.*, 2015), antiviral (El-Sabbagh *et al.*, 2009), anti-tumor (Farag *et al.*, 2010), analgesic, sedative (Karrouchi *et al.*, 2014), antimicrobial (Mert *et al.*, 2014), and anti-inflammatory activities (Alegaon *et al.*, 2014). In addition, pyrazoles have a wide variety of applications in the agrochemical and pharmaceutical industries (Patel *et al.*, 2004). Recently we have reported the synthesis of substituted pyrazoles (Karrouchi *et al.*, 2013). As an extension of our work on the structural characterization of pyrazoles, the title compound was prepared and analysed by single-crystal X-ray diffraction.

The molecule of the title compound is build up from two phenyl rings linked to a pyrazole ring through the carbohydrazide group as shown in Fig. 1. The phenyl rings C7–C12 and C13–C18) are nearly approximately as indicated by the dihedral angle of 78.07 (8)° between them, and form makes dihedral angles of 56.43 (8)° and 24.59 (8)°, respectively, with the pyrazole ring. In the crystal, the molecules held together by N1–H1N···O1 hydrogen bonds and form one-dimensional chains along the [0 1 0] direction.

### S2. Experimental

To a solution of 5-methyl-1*H*-pyrazole-3-carbohydrazide (1 mmol) in 10 ml of ethanol, an equimolar amount of the benzophenon was added in the presence of acetic acid. The mixture was maintained under reflux for 2 h, then the precipitate formed was filtered out washed with ethanol and recrystallized from ethanol. Single crystals of the title compound were obtained on slow evaporation of the solvent (yield 87%; m. p. 595 K).

IR (KBr, ν(cm^-1^)): 3241 (NH), 1655 (C=O), 1592 (N=CH). ^1^*H*-NMR (300 MHz, DMSO-d6, δ (p.p.m.)): δ = 2.19 (s, 3H, –CH3), 6.46 (s, 1H, Pz—H), 7.37–7.56 (m, 10H, Ar—H), 9.80 (s, 1H, N=CH), 11.23 (s, 1H, CONH), 12.99 (s, 1H, Pz—NH). MS: m/*z* = 304.9 (M—H+).

### S3. Refinement

The H atoms were located in a difference Fourier map and treated as riding, with C—H = 0.93-0.96 Å, N—H = 0.86 Å, and with *U*_iso_(H) = 1.2 *U*_eq_ (C, N) or 1.5 *U*_eq_ for methyl H atoms. The reflection (0 0 2) affected by the beamstop was removed during the last cycles of refinement.

### Crystal data

**Table:**

C_18_H_16_N_4_O	*F*(000) = 1280
*M**_r_* = 304.35	*D*_x_ = 1.284 Mg m^−^^3^
Orthorhombic, *P**b**c**a*	Melting point: 595 K
*a* = 11.0299 (2) Å	Mo *K*α radiation, λ = 0.71073 Å
*b* = 14.1131 (2) Å	µ = 0.08 mm^−^^1^
*c* = 20.2211 (3) Å	*T* = 296 K
*V* = 3147.74 (9) Å^3^	Block, colourless
*Z* = 8	0.40 × 0.32 × 0.25 mm

### Data collection

**Table:**

Bruker X8 APEX diffractometer	3117 reflections with *I* > 2σ(*I*)
Radiation source: fine-focus sealed tube	*R*_int_ = 0.029
Graphite monochromator	θ_max_ = 27.9°, θ_min_ = 2.6°
φ and ω scans	*h* = −14→13
31259 measured reflections	*k* = −18→17
3766 independent reflections	*l* = −26→26

### Refinement

**Table:**

Refinement on *F*^2^	0 restraints
Least-squares matrix: full	Hydrogen site location: inferred from neighbouring sites
*R*[*F*^2^ > 2σ(*F*^2^)] = 0.047	H-atom parameters constrained
*wR*(*F*^2^) = 0.136	*w* = 1/[σ^2^(*F*_o_^2^) + (0.0655*P*)^2^ + 1.2766*P*] where *P* = (*F*_o_^2^ + 2*F*_c_^2^)/3
*S* = 1.04	(Δ/σ)_max_ < 0.001
3766 reflections	Δρ_max_ = 0.33 e Å^−^^3^
208 parameters	Δρ_min_ = −0.26 e Å^−^^3^

### Special details

**Table:**

Geometry. All e.s.d.'s (except the e.s.d. in the dihedral angle between two l.s. planes) are estimated using the full covariance matrix. The cell e.s.d.'s are taken into account individually in the estimation of e.s.d.'s in distances, angles and torsion angles; correlations between e.s.d.'s in cell parameters are only used when they are defined by crystal symmetry. An approximate (isotropic) treatment of cell e.s.d.'s is used for estimating e.s.d.'s involving l.s. planes.

### Fractional atomic coordinates and isotropic or equivalent isotropic displacement parameters (Å^2^)

**Table:**

	*x*	*y*	*z*	*U*_iso_*/*U*_eq_	
C1	0.95160 (17)	0.23308 (11)	0.22978 (11)	0.0559 (5)	
H1A	1.0236	0.2614	0.2119	0.084*	
H1B	0.9735	0.1873	0.2628	0.084*	
H1C	0.9073	0.2024	0.1950	0.084*	
C2	0.87443 (13)	0.30798 (9)	0.26034 (8)	0.0351 (3)	
C3	0.88909 (13)	0.40388 (9)	0.26613 (7)	0.0349 (3)	
H3	0.9538	0.4403	0.2512	0.042*	
C4	0.78552 (12)	0.43513 (8)	0.29934 (7)	0.0292 (3)	
C5	0.75426 (13)	0.53261 (9)	0.31897 (7)	0.0304 (3)	
C6	0.50855 (13)	0.62906 (9)	0.40692 (7)	0.0320 (3)	
C7	0.40970 (13)	0.55750 (9)	0.40017 (7)	0.0338 (3)	
C8	0.42278 (16)	0.46563 (11)	0.42431 (8)	0.0444 (4)	
H8	0.4945	0.4480	0.4451	0.053*	
C9	0.33011 (19)	0.40042 (13)	0.41756 (10)	0.0567 (5)	
H9	0.3398	0.3393	0.4339	0.068*	
C10	0.22390 (18)	0.42554 (14)	0.38688 (10)	0.0576 (5)	
H10	0.1616	0.3816	0.3825	0.069*	
C11	0.20965 (16)	0.51595 (14)	0.36252 (10)	0.0537 (4)	
H11	0.1379	0.5327	0.3414	0.064*	
C12	0.30150 (15)	0.58207 (12)	0.36924 (8)	0.0439 (4)	
H12	0.2908	0.6432	0.3530	0.053*	
C13	0.48843 (13)	0.71522 (9)	0.44807 (7)	0.0332 (3)	
C14	0.56815 (15)	0.79196 (10)	0.44439 (8)	0.0413 (4)	
H14	0.6288	0.7925	0.4124	0.050*	
C15	0.55768 (17)	0.86695 (11)	0.48774 (10)	0.0505 (4)	
H15	0.6118	0.9174	0.4852	0.061*	
C16	0.46712 (17)	0.86744 (12)	0.53494 (9)	0.0514 (4)	
H16	0.4611	0.9176	0.5646	0.062*	
C17	0.38608 (17)	0.79383 (13)	0.53797 (9)	0.0498 (4)	
H17	0.3243	0.7948	0.5692	0.060*	
C18	0.39577 (14)	0.71784 (11)	0.49463 (8)	0.0414 (3)	
H18	0.3400	0.6685	0.4968	0.050*	
N1	0.76763 (11)	0.28765 (8)	0.28919 (7)	0.0368 (3)	
H1N	0.7385	0.2312	0.2914	0.044*	
N2	0.71101 (11)	0.36377 (8)	0.31412 (7)	0.0355 (3)	
N3	0.64218 (11)	0.54067 (8)	0.34657 (7)	0.0366 (3)	
HN3	0.5896	0.4961	0.3424	0.044*	
N4	0.61501 (11)	0.62165 (8)	0.38121 (6)	0.0346 (3)	
O1	0.82345 (10)	0.59943 (7)	0.31103 (6)	0.0420 (3)	

### Atomic displacement parameters (Å^2^)

**Table:**

	*U*^11^	*U*^22^	*U*^33^	*U*^12^	*U*^13^	*U*^23^
C1	0.0517 (10)	0.0332 (8)	0.0829 (13)	0.0055 (7)	0.0233 (9)	−0.0125 (8)
C2	0.0336 (7)	0.0267 (6)	0.0450 (8)	0.0025 (5)	0.0051 (6)	−0.0028 (5)
C3	0.0334 (7)	0.0265 (6)	0.0447 (8)	−0.0014 (5)	0.0091 (6)	−0.0007 (5)
C4	0.0298 (6)	0.0212 (6)	0.0367 (7)	0.0000 (5)	0.0014 (5)	−0.0006 (5)
C5	0.0323 (7)	0.0209 (6)	0.0381 (7)	0.0011 (5)	0.0014 (5)	−0.0014 (5)
C6	0.0332 (7)	0.0264 (6)	0.0364 (7)	0.0032 (5)	0.0001 (5)	−0.0003 (5)
C7	0.0340 (7)	0.0313 (6)	0.0362 (7)	0.0004 (5)	0.0043 (6)	−0.0032 (5)
C8	0.0480 (9)	0.0376 (8)	0.0475 (9)	−0.0026 (7)	0.0017 (7)	0.0059 (6)
C9	0.0650 (12)	0.0432 (9)	0.0618 (11)	−0.0147 (8)	0.0084 (9)	0.0086 (8)
C10	0.0510 (11)	0.0598 (11)	0.0621 (11)	−0.0240 (9)	0.0113 (9)	−0.0067 (9)
C11	0.0384 (9)	0.0655 (11)	0.0573 (10)	−0.0062 (8)	−0.0015 (8)	−0.0080 (9)
C12	0.0398 (8)	0.0410 (8)	0.0510 (9)	0.0025 (6)	−0.0023 (7)	−0.0015 (7)
C13	0.0332 (7)	0.0274 (6)	0.0391 (7)	0.0062 (5)	−0.0015 (6)	−0.0017 (5)
C14	0.0401 (8)	0.0320 (7)	0.0519 (9)	0.0034 (6)	0.0059 (7)	−0.0040 (6)
C15	0.0500 (10)	0.0332 (7)	0.0682 (11)	−0.0008 (7)	−0.0002 (8)	−0.0120 (7)
C16	0.0571 (10)	0.0424 (9)	0.0549 (10)	0.0092 (8)	−0.0010 (8)	−0.0187 (7)
C17	0.0507 (10)	0.0510 (9)	0.0477 (9)	0.0078 (7)	0.0097 (8)	−0.0103 (7)
C18	0.0391 (8)	0.0378 (7)	0.0473 (8)	0.0017 (6)	0.0065 (7)	−0.0044 (6)
N1	0.0342 (6)	0.0183 (5)	0.0580 (8)	−0.0004 (4)	0.0070 (6)	−0.0042 (5)
N2	0.0318 (6)	0.0208 (5)	0.0540 (7)	−0.0002 (4)	0.0077 (5)	−0.0035 (5)
N3	0.0325 (6)	0.0226 (5)	0.0547 (7)	−0.0017 (4)	0.0064 (5)	−0.0095 (5)
N4	0.0341 (6)	0.0237 (5)	0.0459 (7)	0.0029 (4)	0.0022 (5)	−0.0067 (5)
O1	0.0402 (6)	0.0216 (5)	0.0643 (7)	−0.0040 (4)	0.0108 (5)	−0.0039 (4)

### Geometric parameters (Å, º)

**Table:**

C1—C2	1.491 (2)	C10—C11	1.377 (3)
C1—H1A	0.9600	C10—H10	0.9300
C1—H1B	0.9600	C11—C12	1.384 (2)
C1—H1C	0.9600	C11—H11	0.9300
C2—N1	1.3455 (19)	C12—H12	0.9300
C2—C3	1.3681 (18)	C13—C18	1.390 (2)
C3—C4	1.3966 (19)	C13—C14	1.397 (2)
C3—H3	0.9300	C14—C15	1.379 (2)
C4—N2	1.3339 (16)	C14—H14	0.9300
C4—C5	1.4727 (17)	C15—C16	1.382 (3)
C5—O1	1.2238 (16)	C15—H15	0.9300
C5—N3	1.3611 (18)	C16—C17	1.372 (3)
C6—N4	1.2883 (19)	C16—H16	0.9300
C6—C13	1.4901 (18)	C17—C18	1.389 (2)
C6—C7	1.4924 (19)	C17—H17	0.9300
C7—C12	1.391 (2)	C18—H18	0.9300
C7—C8	1.393 (2)	N1—N2	1.3411 (15)
C8—C9	1.382 (2)	N1—H1N	0.8600
C8—H8	0.9300	N3—N4	1.3736 (15)
C9—C10	1.372 (3)	N3—HN3	0.8600
C9—H9	0.9300		
			
C2—C1—H1A	109.5	C10—C11—C12	120.42 (17)
C2—C1—H1B	109.5	C10—C11—H11	119.8
H1A—C1—H1B	109.5	C12—C11—H11	119.8
C2—C1—H1C	109.5	C11—C12—C7	120.26 (16)
H1A—C1—H1C	109.5	C11—C12—H12	119.9
H1B—C1—H1C	109.5	C7—C12—H12	119.9
N1—C2—C3	106.10 (12)	C18—C13—C14	118.57 (13)
N1—C2—C1	121.89 (13)	C18—C13—C6	120.63 (13)
C3—C2—C1	132.01 (14)	C14—C13—C6	120.61 (13)
C2—C3—C4	104.89 (12)	C15—C14—C13	120.56 (15)
C2—C3—H3	127.6	C15—C14—H14	119.7
C4—C3—H3	127.6	C13—C14—H14	119.7
N2—C4—C3	111.92 (11)	C14—C15—C16	120.23 (16)
N2—C4—C5	120.04 (12)	C14—C15—H15	119.9
C3—C4—C5	128.03 (12)	C16—C15—H15	119.9
O1—C5—N3	123.76 (12)	C17—C16—C15	119.89 (15)
O1—C5—C4	122.58 (12)	C17—C16—H16	120.1
N3—C5—C4	113.66 (11)	C15—C16—H16	120.1
N4—C6—C13	115.30 (12)	C16—C17—C18	120.41 (16)
N4—C6—C7	125.03 (12)	C16—C17—H17	119.8
C13—C6—C7	119.64 (12)	C18—C17—H17	119.8
C12—C7—C8	118.58 (14)	C17—C18—C13	120.29 (15)
C12—C7—C6	119.94 (13)	C17—C18—H18	119.9
C8—C7—C6	121.48 (14)	C13—C18—H18	119.9
C9—C8—C7	120.57 (16)	N2—N1—C2	113.57 (11)
C9—C8—H8	119.7	N2—N1—H1N	123.2
C7—C8—H8	119.7	C2—N1—H1N	123.2
C10—C9—C8	120.26 (17)	C4—N2—N1	103.52 (11)
C10—C9—H9	119.9	C5—N3—N4	118.48 (11)
C8—C9—H9	119.9	C5—N3—HN3	120.8
C9—C10—C11	119.91 (16)	N4—N3—HN3	120.8
C9—C10—H10	120.0	C6—N4—N3	118.18 (12)
C11—C10—H10	120.0		

### Hydrogen-bond geometry (Å, º)

**Table:**

*D*—H···*A*	*D*—H	H···*A*	*D*···*A*	*D*—H···*A*
N1—H1*N*···O1^i^	0.86	2.02	2.8740 (15)	172

Symmetry code: (i) −*x*+3/2, *y*−1/2, *z*.
