# Supplementary figures and images for: Crystal structure of N′-di­phenyl­methyl­idene-5-methyl-1H-pyrazole-3-carbo­hydrazide
Source: Acta Crystallogr E Crystallogr Commun. 2015 Oct 28;71(Pt 11):o890–1. doi: 10.1107/S2056989015020071 (PMC4645091; doi:10.1107/S2056989015020071)

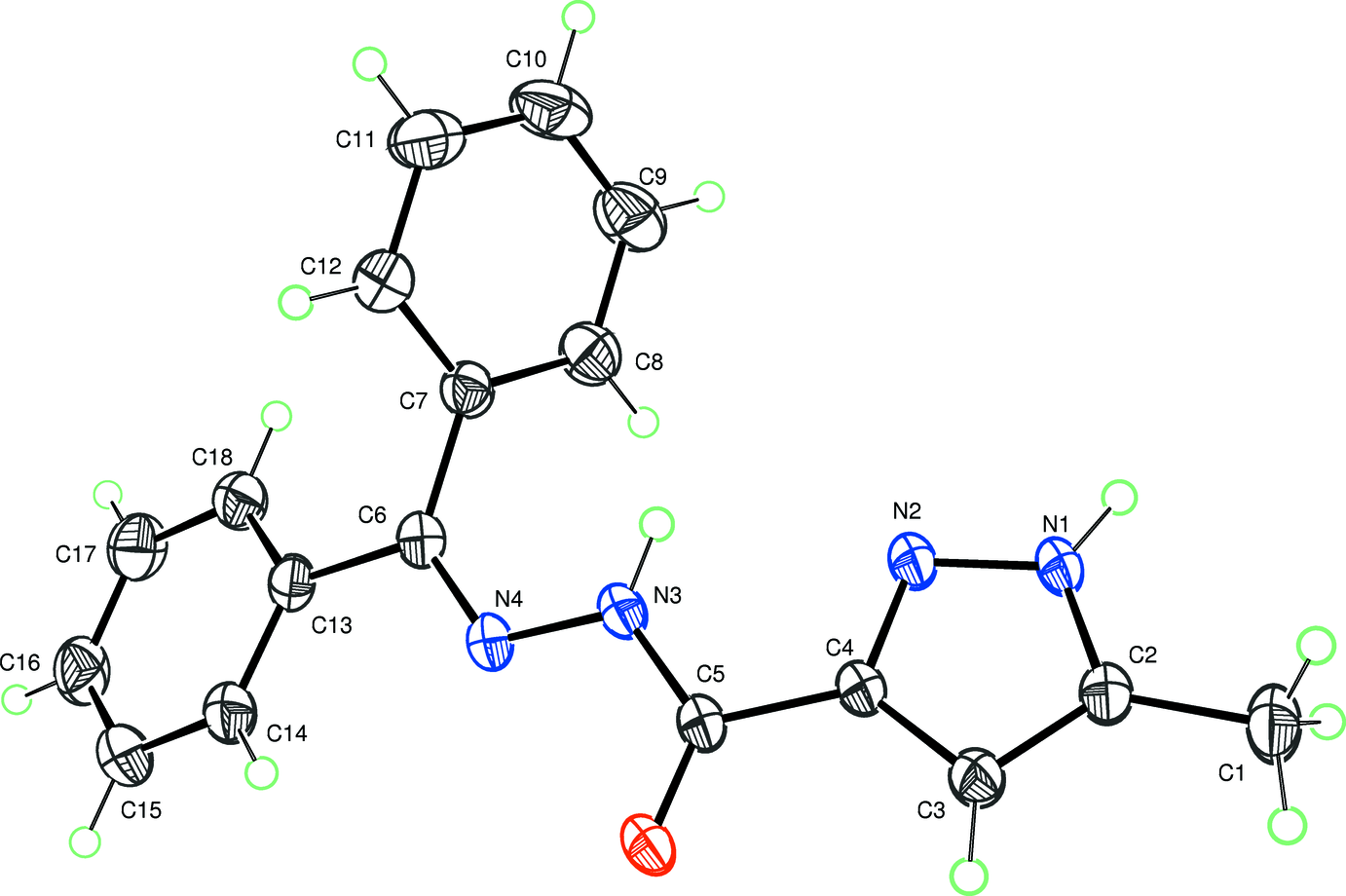

Supplement: Supplementary file 4 [file e-71-0o890-fig1.tif]

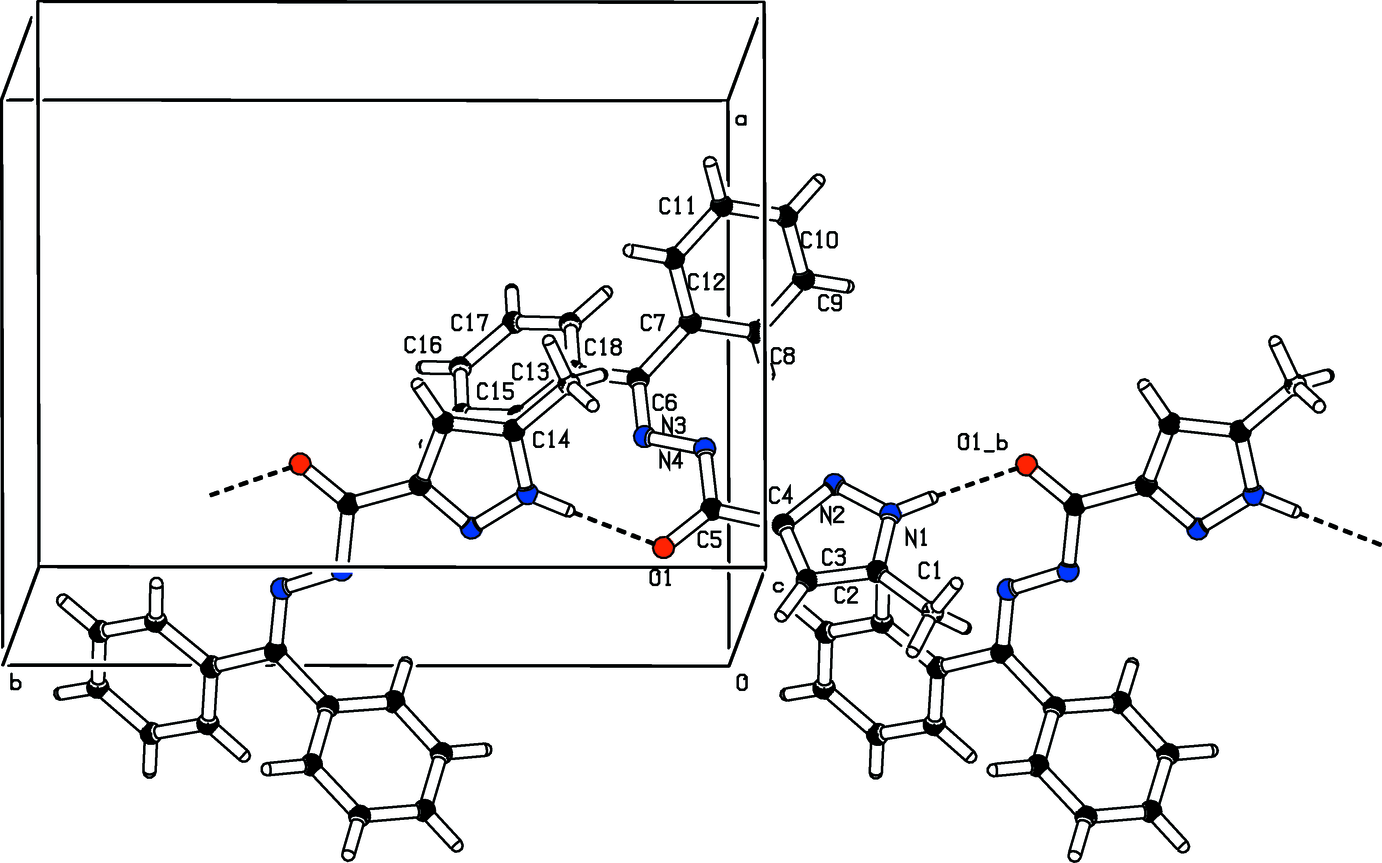

Supplement: Supplementary file 5 [file e-71-0o890-fig2.tif]
